# Investigation of the potential association between the use of fluoxetine and occurrence of acute pancreatitis: a Danish register-based cohort study

**DOI:** 10.1093/ije/dyac071

**Published:** 2022-04-26

**Authors:** Mia Aakjær, Sarah Brøgger Kristiansen, Kathrine Pape, Maurizio Sessa, Kim Peder Dalhoff, Marie Louise De Bruin, Morten Andersen

**Affiliations:** Pharmacovigilance Research Center, Department of Drug Design and Pharmacology, University of Copenhagen, Copenhagen, Denmark; Pharmacovigilance Research Center, Department of Drug Design and Pharmacology, University of Copenhagen, Copenhagen, Denmark; Pharmacovigilance Research Center, Department of Drug Design and Pharmacology, University of Copenhagen, Copenhagen, Denmark; Pharmacovigilance Research Center, Department of Drug Design and Pharmacology, University of Copenhagen, Copenhagen, Denmark; Department of Clinical Pharmacology, Bispebjerg and Frederiksberg Hospital, Copenhagen, Denmark; Department of Clinical Medicine, University of Copenhagen, Copenhagen, Denmark; Copenhagen Centre for Regulatory Science (CORS), Department of Pharmacy, University of Copenhagen, Copenhagen, Denmark; Utrecht Institute for Pharmaceutical Sciences (UIPS), Division of Pharmacoepidemiology and Clinical Pharmacology, Utrecht University, Utrecht, The Netherlands; Pharmacovigilance Research Center, Department of Drug Design and Pharmacology, University of Copenhagen, Copenhagen, Denmark

**Keywords:** Selective serotonin reuptake inhibitors, fluoxetine, acute pancreatitis, drug safety

## Abstract

**Background:**

There is currently conflicting evidence of the association between the use of selective serotonin reuptake inhibitors (SSRIs) and acute pancreatitis. The SSRI fluoxetine has been suspected to be the driver of this serious outcome. Therefore, this study aims to investigate the potential association between fluoxetine use and the occurrence of acute pancreatitis.

**Methods:**

We conducted a nationwide cohort study using Danish register-based data from 1996 to 2016. The exposed group were new users of fluoxetine (1-year washout). The control subjects were new users of citalopram or SSRIs, excluding fluoxetine. The outcome was an incident diagnosis of acute pancreatitis with a 5-year washout. We used an intention-to-treat approach following patients for a maximum of 6 months. Cox regression analyses were performed, estimating hazard ratios (HRs) and 95% confidence intervals (CIs) adjusted for age/sex, comorbidities and co-medications, using propensity score adjustment and matching.

**Results:**

In the propensity score-matched analyses, 61 783 fluoxetine users were included. The incidence rates among users of fluoxetine and other SSRIs were 5.33 (3.05–8.66) and 5.36 (3.06–8.70) per 10 000 person-years, respectively. No increased risk of acute pancreatitis was identified following fluoxetine exposure compared with either citalopram [HR 1.00, 95% CI 0.50–2.00) or other SSRIs (0.76, 0.40–1.46).

**Conclusions:**

Fluoxetine use was not associated with an increased risk of acute pancreatitis compared with citalopram or other SSRIs. The absolute risk of acute pancreatitis was low and did not vary between different SSRIs. Further research is needed to determine whether there is a class effect on the risk of acute pancreatitis.

Key messages

- Our study did not identify an increased risk of acute pancreatitis among new users of fluoxetine compared with users of citalopram or other selective serotonin reuptake inhibitors (SSRIs).
- Sensitivity analyses including recurrent events of acute pancreatitis showed higher point estimates and including recurrent events may bias the results.
- Further research is needed to determine whether SSRIs have a class effect on the risk of acute pancreatitis.

## Background

Acute pancreatitis is one of the most common gastrointestinal conditions leading to hospital admission, with an incidence rate (IR) of 33.7 [95% confidence interval (CI) 23.3–48.8] per 100 000 person-years.^1^ The most common risk factors are alcohol abuse and gallstones.^2^^,^^3^ The procedure endoscopic retrograde cholangiopancreatography (ERCP) and comorbidities such as hypertriglyceridemia and diabetes mellitus are other known risk factors.^3^ Drug-induced pancreatitis is considered the third most common cause following alcohol abuse and gallstones, even though it is a rare event.^2^ According to a report from the World Health Organization (WHO), more than 525 drugs, including azathioprine, metronidazole and valproic acid, were suspected to induce acute pancreatitis between 1968 and 1993.^2^ However, information on risks associated with individual products today is still sparse, and in many cases, evidence is based on case reports.^2^ In 1993, the WHO reported a signal linking the selective serotonin reuptake inhibitor (SSRI) fluoxetine to pancreatitis (Uppsala Monitoring Centre, Pinelopi Lundquist, personal communication).^4^ To our knowledge, only a single observational study has investigated the risk of acute pancreatitis among fluoxetine users. Lancashire *et al*.^5^ found an odds ratio (OR) of 1.6 (95% CI 0.8–3.2). Since then, three case-control studies have investigated the association between SSRIs as a drug class and the risk of acute pancreatitis, but with inconsistent results.^6–8^ Two recent meta-analyses evaluated the potential association between SSRIs and acute pancreatitis based on these studies. Yao *et al*. included all four case-control studies, whereas Lai *et al*. included the three studies investigating only SSRIs. Both meta-analyses highlight the potential increased risk of acute pancreatitis following SSRI exposure, and stress the need for more evidence to claim causation.^9^^,^^10^ Yao *et al*.^9^ highlight the need for larger and more high-quality studies to determine which SSRIs trigger acute pancreatitis and at what dosing regimen. Lai *et al*.^10^ call for case reports with a re-challenge test, more real-world data and animal studies. In a pilot signal detection study using sequential analysis, we identified an association between fluoxetine use and the occurrence of acute pancreatitis, with a hazard ratio (HR) of 1.5, 95% CI 1.1–2.1.^11^ In a subsequent study with improved analysis choices, however, the potential safety signal was not confirmed.^12^ In surveillance studies investigating a large number of outcomes, it is not possible to adjust for confounders tailored to each outcome. Rather, it is common to adjust for a number of general confounding factors.^13^ Therefore, potential signals should be followed up with a study using tailored confounding adjustment. Due to the inconsistent results and since acute pancreatitis is associated with high morbidity and mortality,^14^ we wished to further investigate the association between fluoxetine use and acute pancreatitis by adjusting for tailored confounding in different ways, rather than general confounding, and to check the robustness of the findings by investigating analysis choices that differed in existing studies, e.g. which ICD-10 codes defined the outcome, and definitions of washout and follow-up periods.

## Methods

### Study population and design

**Figure 1:**
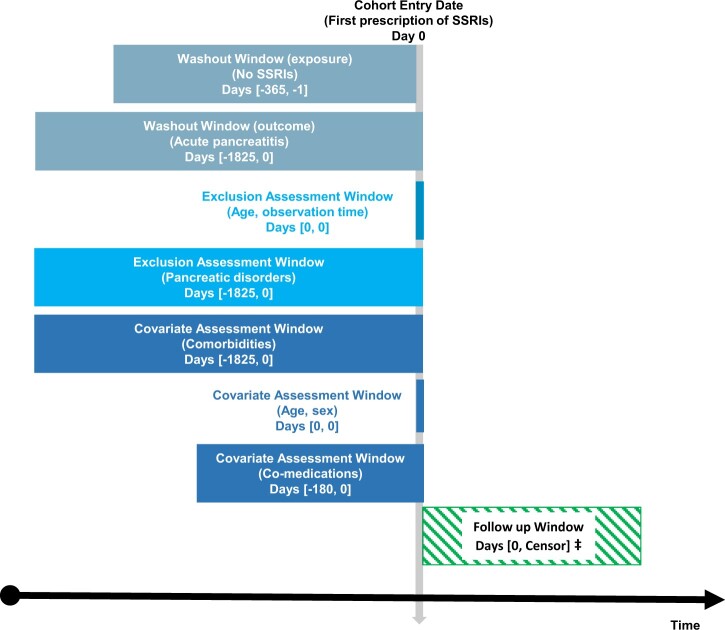
Graphical depiction of the study design with cohort entry, assessment windows for exclusions, washout for the exposure and outcome, covariates assessment window for comorbidities and co-medications, and follow-up window. Figure based on Schneeweiss *et al.*^18^ Licensed under CC BY, can be found at [https://presc.sdu.dk/repeat-diagrams/]. ‡Earliest of outcome of interest (acute pancreatitis), death, emigration, exclusion criterion, 180 days of follow-up, or end of study period. SSRIs, selective serotonin reuptake inhibitors

We included a cohort of new SSRI users [Anatomical Therapeutic Chemical (ATC) Classification: N06AB] identified between 1 January 1997 and 31 December 2016, on the date of their first drug dispensing at the pharmacy (the index date) who had not been dispensed SSRIs during the previous year (1-year washout period). We excluded patients who: (i) had more than one SSRIs dispensed on the index date; (ii) were younger than 18 years on the index date; (iii) were not resident in Denmark on the index date; (iv) had less than 1 year of observation time before the index date; (v) had a previous diagnosis of acute pancreatitis, chronic pancreatitis, other pancreatic disorders or pancreatic cancer 5 years before the index date; and (vi) had a previous diagnosis of human immunodeficiency virus (HIV) infection,^15^ or a procedure code for ERCP^16^ 5 years before the index date, or fill of a prescription of azathioprine or the prodrug mercaptopurine^17^ 6 months before the index date. Definitions of exclusion criteria are provided in Supplementary Table S1 (available as Supplementary data at *IJE* online). Figure 1 shows a graphical depiction of the study design.

### Data sources

We used Danish health care registry data. The unique personal identification number (CPR number), assigned to all residents at birth or immigration, enables merging information from different registers.^19^ We included data from the Danish National Prescription Registry, providing data on all prescription drugs dispensed to Danish residents in community pharmacies.^20^ This registry was used to identify exposure, comparators and co-medications. The Danish National Patient Register contains data on somatic and psychiatric in- and outpatient diagnoses in all hospitals, coded as International Statistical Classification of Diseases and Related Health Problems (ICD) 8th revision until 1994 and 10th revision onwards.^21^ This registry was used to identify admissions due to acute pancreatitis, exclusion criteria and comorbidities. Furthermore, the Cause of Death Register^22^ and the Central Person Registry containing data on vital status and migration^23^ were used to identify whether the patients died or migrated during the study period, respectively.

### Exposures

The exposed group was patients who redeemed a prescription of fluoxetine (ATC: N06AB03). In the primary analysis, we compared fluoxetine with citalopram users. In the secondary analysis, the comparison group was users of SSRIs, excluding fluoxetine, referred to as other SSRIs. Patients were followed using an intention-to-treat approach for a maximum of 6 months from the index date to the occurrence of an event of interest, study end or censoring. Patients were censored if they: (i) experienced an exclusion criterion after the index date; (ii) died; (iii) emigrated from Denmark, whatever came first.

### Outcome

The outcome was an incident diagnosis of acute pancreatitis with an event-free washout period of 5 years before the index date (Supplementary Table S1, available as Supplementary data at *IJE* online). Data from inpatient admissions and primary diagnoses were used to identify the outcome.

### Covariates

Potential confounders, including both comorbidities and co-medications, were identified from recent review papers on acute pancreatitis and drug-induced acute pancreatitis.^2^^,^^3^^,^^24^^,^^25^ Comorbidities and their drug proxies included gallstones,^26^ alcohol-related diseases and medication against alcohol dependence,^26^ smoking-related diseases and medications to treat chronic obstructive pulmonary disease,^27^ hyperlipidaemia and statin use,^28^ inflammatory bowel disease,^29^ diabetes and antidiabetic medications,^30^ obesity and anti-obesity medications^31^ and ischaemic heart disease.^32^ Co-medications included calcium channel blockers,^33^ non-steroidal anti-inflammatory drugs (NSAIDs) excluding cyclooxygenase-2 (COX-2) inhibitors,^34^ valproic acid,^35^ angiotensin-converting enzyme (ACE) inhibitors,^33^^,^^35^ metronidazole^36^ and oral glucocorticosteroids.^27^ The definition of co-medication covariates corresponded to at least one dispensing of a co-medication within 6 months before the index date. Moreover, we used restriction (exclusion criterion 6) on risk factors that were too rare to be included in the propensity score.

### Statistical analysis

IRs were calculated with 95% CIs based on the gamma inverse cumulative distribution function. We used Cox regression models to estimate crude and adjusted HRs and 95% CIs for the study outcome. To control for confounding, we performed an age- and sex-adjusted analysis, a propensity score-adjusted analysis and an analysis using propensity score matching. The propensity score was based on risk factors for the outcome covering age,^2^ sex,^2^ comorbidities and co-medications. Logistic regression was used to estimate the propensity score stratified by index year, with exposure as the dependent variable and covariates as independent variables.^37^ The propensity score was stratified into quintiles within the index year.^38^ Propensity score matching was performed 1:1 using the greedy nearest neighbour method with no replacement and a maximum caliper distance of 0.2 of the logit of the propensity score. The matching was performed exactly on index year. We calculated standardized mean differences to assess the covariate balance post-matching. Values below 0.25 were considered well balanced.^38^

We tested for violations of the proportional hazard assumption by including interaction terms between each risk factor and time in the model. A separate test for proportional hazards was performed, including all covariates and time-interacting terms.^39^

### Sensitivity analyses

We conducted eight sensitivity analyses (analysis S1-S8) of each exposure-comparator set. In analysis S1-S3, we modified the definition of the outcome. In S1, we included secondary diagnoses in addition to the main diagnosis (regarded as the cause of admission). In analysis S2, the admission type included outpatient visits in addition to inpatient hospitalizations. In analysis S3, we excluded ICD-10 codes representing gallstone (K85.1) and alcohol-induced acute pancreatitis (K85.2). In analysis S4, the length of the follow-up was reduced from 6 to 3 months. In analysis S5, we allowed patients to have recurrent events of acute pancreatitis to emulate our pilot study which allowed inclusion of patients with previous events.^11^ In analysis S6, we extended the washout period of the outcome by including all available information in the data. In analysis S7, patients who filled a prescription of another SSRI during the 6 months follow-up were considered as switchers and censored at the time of switching. Finally, in analysis S8, the washout of the exposure was extended from 1 year to 2 years (Supplementary Table S2, available as Supplementary data at *IJE* online).

## Results

### Cohort characteristics

**Figure 2:**
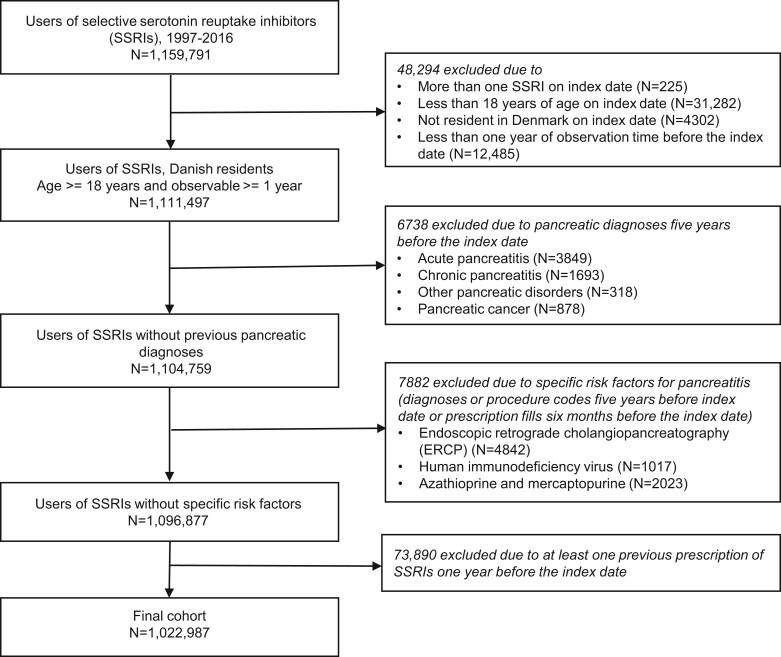
Study flowchart. SSRIs, selective serotonin reuptake inhibitors

In total 1 022 987 new users of SSRIs were included in the final cohort after applying exclusion criteria (Figure 2). We identified 61 783 new users of fluoxetine (Table 1). The reference groups consisted of 577 573 and 961 204 users of citalopram and other SSRIs in the unmatched cohorts, respectively. More information about cohort characteristics is provided in Tables 1 and 2. In the propensity score-matched cohorts, 61 783 fluoxetine, citalopram and other SSRI users were included. The distribution of baseline covariates in these cohorts was similar, with the highest standardized differences of 1.59% (Tables 1 and 2).

**Table 1:** Cohort characteristics of the fluoxetine users compared with citalopram users

	Unmatched cohort			Matched cohort		
Variables	Fluoxetine (*n* = 61 783)	Citalopram (*n* = 577 573)	Standardized difference	Fluoxetine (*n* = 61 783)	Citalopram (*n* = 61 783)	Standardized difference
	*n* (%)	*n* (%)	%	*n* (%)	*n* (%)	*n* (%)
Sex, female	38 431 (62.2)	347 878 (60.2)	4.05	38 431 (62.2)	38 456 (62.2)	0.08
Age, mean (SD)	47.2 (17.6)	55.2 (21.0)	40.82	47.2 (17.6)	47.3 (17.7)	0.44
Comorbidities						
Gallstones	729 (1.18)	7663 (1.33)	1.32	729 (1.18)	690 (1.12)	0.59
Alcohol	3710 (6.00)	26 264 (4.55)	6.52	3710 (6.00)	3613 (5.85)	0.66
Smoking	5624 (9.10)	59 590 (10.32)	4.10	5624 (9.10)	5482 (8.87)	0.80
Hyperlipideamia	2050 (3.32)	62 798 (10.87)	29.75	2050 (3.32)	1986 (3.21)	0.58
IBD	329 (0.53)	3639 (0.63)	1.28	329 (0.53)	295 (0.48)	0.78
Diabetes mellitus	2151 (3.48)	36 495 (6.32)	13.17	2151 (3.48)	2014 (3.26)	1.23
Obesity	2808 (4.54)	17 265 (2.99)	8.18	2808 (4.54)	2607 (4.22)	1.59
IHD	2241 (3.63)	45 256 (7.32)	15.94	2241 (3.63)	2194 (3.55)	0.41
Co-medications						
CCB	3150 (5.10)	63 050 (10.20)	19.44	3150 (5.10)	3073 (4.97)	0.57
NSAIDs	11 253 (18.2)	112 385 (18.19)	0.29	11 253 (18.2)	11 113 (17.99)	0.59
Valproic acid	166 (0.27)	3862 (0.63)	5.25	166 (0.27)	137 (0.22)	0.95
ACE inhibitors	2600 (4.21)	59 136 (9.57)	22.05	2600 (4.21)	2530 (4.09)	0.57
Metronidazole	1026 (1.66)	8529 (1.38)	2.04	1026 (1.66)	953 (1.54)	0.94
Glucocorticosteroids	2591 (4.19)	41 745 (7.23)	7.68	2591 (4.19)	2487 (4.03)	0.85

ACE, angiotensin-converting enzyme; CCB, calcium channel blockers; COPD, chronic obstructive pulmonary disease; IBD, inflammatory bowel disease; IHD, ischaemic heart disease; NSAIDs, non-steroidal anti-inflammatory drugs; SD, standard deviation.

**Table 2:** Cohort characteristics of the fluoxetine users compared with users of other SSRIs

	Unmatched cohort			Matched cohort		
Variables	Fluoxetine (*n* = 61 783) *n* (%)	Other SSRIs^a^ (*n* = 961 204) *n* (%)	Standardized difference %	Fluoxetine (n = 61 783) *n* (%)	Other SSRIs^a^ (*n* = 61 783) *n* (%)	Standardized difference %
Sex, female	38 431 (62.2)	579 429 (60.3)	3.94	38 431 (62.2)	38 438 (62.2)	0.02
Age, mean (SD)	47.2 (17.6)	52.7 (20.7)	28.27	47.2 (17.6)	47.3 (17.7)	0.37
Comorbidities						
Gallstones	729 (1.18)	12 458 (1.30)	1.05	729 (1.18)	687 (1.11)	0.64
Alcohol	3710 (6.00)	43 431 (4.52)	6.66	3710 (6.00)	3652 (5.91)	0.40
Smoking	5624 (9.10)	93 560 (9.73)	2.16	5624 (9.10)	5562 (9.00)	0.35
Hyperlipidaemia	2050 (3.32)	96 378 (10.03)	27.13	2050 (3.32)	1993 (3.23)	0.52
IBD	329 (0.53)	5954 (0.62)	1.15	329 (0.53)	298 (0.48)	0.71
Diabetes mellitus	2151 (3.48)	54 477 (5.67)	10.48	2151 (3.48)	2028 (3.28)	1.10
Obesity	2808 (4.54)	30 109 (3.13)	7.36	2808 (4.54)	2664 (4.31)	1.13
IHD	2241 (3.63)	61 074 (6.35)	12.55	2241 (3.63)	2152 (3.48)	0.78
Co-medications						
CCB	3150 (5.10)	88 351 (9.19)	15.94	3150 (5.10)	3033 (4.91)	0.87
NSAIDs	11 253 (18.2)	168 911 (17.6)	1.67	11 253 (18.2)	11 147 (18.04)	0.45
Valproic acid	166 (0.27)	5326 (0.55)	4.46	166 (0.27)	145 (0.23)	0.68
ACE inhibitors	2600 (4.21)	83 735 (8.71)	18.40	2600 (4.21)	2541 (4.11)	0.48
Metronidazole	1026 (1.66)	14 090 (1.47)	1.57	1026 (1.66)	942 (1.52)	1.09
Glucocorticosteroids	2591 (4.19)	51 175 (5.32)	5.31	2591 (4.19)	2501 (4.05)	0.73

ACE, angiotensin-converting enzyme; CCB, calcium channel blockers; COPD, chronic obstructive pulmonary disease; IBD, inflammatory bowel disease; IHD, ischaemic heart disease; NSAIDs, non-steroidal anti-inflammatory drugs; SD, standard deviation.

a Other SSRIs: all selective serotonin-reuptake inhibitors excluding fluoxetine.

We identified 16 events of acute pancreatitis among users of fluoxetine, corresponding to a crude IR of 5.33 (3.05–8.66) per 10 000 person-years in both the unmatched and matched cohorts (Table 3). In sensitivity analysis S5, allowing recurrent acute pancreatitis events, 26 events of acute pancreatitis were identified among fluoxetine users in the matched and unmatched cohorts.

**Table 3:** Number of events and incidence rate [95% confidence interval (CI)] per 10 000 person-years in new users of fluoxetine, citalopram and selective serotonin reuptake inhibitors excluding fluoxetine (other SSRIs)

	Number of events	Incidence rate (95% CI) per 10 000 person-years
Unmatched cohort		
Fluoxetine	16	5.33 (3.05-8.66)
Citalopram	177	6.45 (5.54-7.48)
Other SSRIs	284	6.21 (5.51-6.98)
Matched cohort		
Fluoxetine	16	5.33 (3.05-8.66)
Citalopram	16	5.36 (3.06-8.70)
Other SSRIs	16	5.36 (3.06-8.70)

SSRIs, selective serotonin reuptake inhibitors.

### Fluoxetine versus citalopram and other selective serotonin reuptake inhibitors

There was no increased relative risk observed for the association between acute pancreatitis and fluoxetine compared with citalopram (propensity score-matched HR 1.00, 0.50–2.00) or other SSRIs (0.76, 0.40–1.46). The age- and sex-adjusted, the propensity score-adjusted and the matched analysis showed comparable results (Table 4).

**Table 4:** Crude hazard ratios (HRs) and 95% confidence intervals (CIs) and age- and sex-adjusted, propensity score-adjusted and matched estimates comparing fluoxetine users with users of citalopram and with selective serotonin reuptake inhibitors excluding fluoxetine (other SSRIs)

	Crude	Age- and sex-adjusted	Propensity score-adjusted	Propensity score matching
Fluoxetine vs citalopram	0.83 (0.50-1.38)	0.95 (0.56-1.58)	0.92 (0.54-1.57)	1.00 (0.50-2.00)
Fluoxetine vs other SSRIs	0.86 (0.52-1.42)	0.95 (0.58-1.58)	0.96 (0.58-1.62)	0.76 (0.40-1.46)

SSRIs, selective serotonin reuptake inhibitors.

### Sensitivity analyses

Sensitivity analyses showed consistent results comparing fluoxetine use with both citalopram use and use of other SSRIs. Analysis S1, including secondary diagnoses in addition to the primary in the definition of acute pancreatitis, analysis S4, with reduced follow-up to 3 month, and analysis S5, that allowed inclusion of recurrent pancreatitis events, provided the highest point estimates when comparing fluoxetine use with both citalopram use and use of other SSRIs (Supplementary Figures S1 and S2, available as Supplementary data at *IJE* online).

## Discussion

In this population-based cohort study, we could not support the hypothesis of an increased risk of acute pancreatitis among new users of fluoxetine compared with new users of citalopram or other SSRIs. Sensitivity analyses provided similar results. However, higher point estimates were observed in analyses including secondary diagnoses, recurrent pancreatitis events and reduced follow-up period. The IR of acute pancreatitis was 5.33 (3.05–8.66) and 5.36 (3.06–8.70) per 10 000 person-years among new users of fluoxetine and other SSRIs in the matched cohorts, respectively. Thus, the absolute risk was low and did not vary between different SSRIs.

Previous studies also have investigated the possible association between SSRI use and acute pancreatitis. A study of spontaneous reports from 2003, investigating SSRIs and risk of hepatic injury and pancreatitis, did not identify an association but highlight that their negative finding does not necessarily exclude the possibility of an increased risk.^4^ In 2003, a study investigated drug-induced acute pancreatitis among a wide range of drugs. They estimated an OR matched for age, sex and general practice of 1.6 (0.8–3.2) among recent users of fluoxetine.^5^ The remaining studies have investigated the risk of SSRIs as a drug class compared with controls from the general population. A regional Danish study from 2007 found an increased risk of acute pancreatitis of 20% among SSRI users. However, the authors suggest that the underlying indication or residual confounding, such as smoking and other lifestyle factors, may explain the increased risk.^6^ In 2012, a Swedish study found no increased risk (OR 1.1, 1.0–1.3) of acute pancreatitis among users of SSRI, after adjusting for confounding factors.^7^ A more recent study from 2017 from Taiwan revealed an adjusted OR of 1.7, 1.2–2.5, among patients with current SSRI use. However, the risk decreased in patients with late use (last tablet more than 8 days before the diagnosis) of SSRIs (OR 1.0, 0.9–1.2).^8^

Our study does not support the hypothesis of an increased pancreatitis risk following the initiation of fluoxetine compared with either citalopram or other SSRIs. However, we cannot exclude an elevated risk below the upper limits of the CIs (2.00 and 1.46 in the propensity score-matched analyses, respectively). We observed differences in analytical methods between existing studies, e.g. differences in ICD-10 codes defining the outcome. Therefore, we conducted sensitivity analyses (S1-S8). In analyses S1 and S2, the outcome definitions additionally included secondary diagnoses and outpatient visits, respectively. Both of these analyses showed similar results. We also conducted a sensitivity analysis excluding diagnoses of gallstone and alcohol-induced acute pancreatitis (analysis S3). However, in our data source, most acute pancreatitis events appeared in the unspecified category (K85.9), and hence no difference was identified. In analysis S4, we reduced the follow-up from 6 to 3 months since the latency period (period from exposure to disease induction^40^) was found to have large variations in a study investigating sertraline-induced acute pancreatitis.^41^ We assumed that patients are more likely to be treated in the first part of the follow-up period, as we used an intention-to-treat approach following patients from the first prescription fill. Since we were only interested in the initial period after exposure, a time-dependent exposure model would not provide any additional information, as most SSRI prescriptions are expected to cover 100 days of drug use. In analysis S5, recurrent events of the outcome were included to emulate our pilot study that allowed inclusion of patients with previous pancreatitis events, since this study detected the association. In this analysis, the point estimates were higher and based on 26 events of acute pancreatitis compared with 16 in the main analysis. We believe that the inclusion of recurrent acute pancreatitis events in the pilot study may explain why we initially identified an increased risk of acute pancreatitis among users of fluoxetine. Recurrence is a well-known condition that occurs in approximately 20–30% of patients with acute pancreatitis.^24^^,^^30^ In analysis S6, all available information in the data was used to define the washout of acute pancreatitis instead of limiting it to 5 years as in the main analysis. Only a few patients were excluded due the extended washout period and therefore the results were similar. In analysis S7, patients who filled a prescription of another SSRI during the 6 months of follow-up were censored. This analysis did not change the results. Finally, we used a washout of the exposure of 1 year: therefore we conducted a sensitivity analysis using 2 years of exposure washout instead. This analysis showed results consistent with the main analysis. In general, the chosen analytical methods did not change the results to any major degree. However, we are not aware of how these could differ in other settings or studies.

A major strength of our study was using a nationwide, well-defined population covering all SSRI users for 20 years. Moreover, we controlled for a large set of potential confounders, including both comorbidities and co-medications. We did this with propensity score adjustment and matching. Finally, we tested the robustness of our findings in eight different sensitivity analyses. We believe that our study results are generalizable, especially to countries with similar health care systems and prescription patterns.

Our study also has some important limitations. First, as a general limitation of dispensing data, we measured redeemed prescriptions and not SSRI intake. We believe, however, that the potential exposure misclassification is non-differential, meaning that there is no difference in actual SSRI intake between the individual drugs. Also, we followed up for a maximum of 6 months after a prescription redemption covering approximately 100 days of treatment. Thus, although a bias towards the null cannot be excluded, the misclassification is considered of minor importance.

Second, we cannot exclude that there is misclassification of outcome, since we could only capture the occurrence of acute pancreatitis that leads to hospital admission (or outpatient contact in analyses S2) and not less severe cases from primary care. However, we believe that this potential misclassification is non-differential, since there is no general concern for acute pancreatitis among users of specific SSRIs. Similarly, we were only able to capture cases of gallstones leading to hospital admission. Also, the covariates representing lifestyle factors, i.e. alcohol-related diseases, smoking-related diseases and obesity, were available as diagnoses rather than clinical measures such as alcohol habits, pack-years and body mass index. By using active comparators we believe that this potential confounding is reduced, since the choice of a specific SSRI is unlikely to be related to lifestyle factors. However, to get wider coverage of the covariates in the registers, we included drug proxies, e.g. anti-obesity medications and medications for treating chronic obstructive pulmonary disease. Nevertheless, our lack of information on covariates causes misclassification of confounders, so residual confounding cannot be ruled out.

Also, unmeasured confounding variables, such as genetic factors and hypercalcaemia,^35^^,^^42^^,^^43^ and exclusion of potential risk factors for acute pancreatitis which we decided not to include due to lack of evidence, such as autoimmune causes, oral contraceptives, hormonal replacement therapy, chronic kidney disease, hepatitis C and hyperparathyroidism,^3^^,^^8^^,^^24^^,^^25^^,^^35^ may have affected our results. Furthermore, confounding by indication may threaten the validity of our results. We were able to control for a large set of potential confounders using propensity scores. This only affected the estimates to a smaller extent and did not affect the overall conclusions (age- and sex-adjusted HR of 0.95, 0.56–1.58, and propensity score-matched HR of 1.00, 0.50–2.00, in the primary analysis). However, we experienced only a few events of the outcome. Therefore, we suggest that future studies allow repeated exposure^44^ or are conducted in a multicountry database.

Finally, we reused our data source from our pilot study. We are aware that it would be preferable to use an external data source. However, Wang *et al*.^45^ highlight situations in which data source reuse is appropriate among other safety surveillance studies using sequential analysis similar to our approach in the pilot study. We re-evaluated the association by using tailored rather than general confounder adjustment that applies to several outcomes. Furthermore, we conducted sensitivity analyses to ensure the robustness of our findings. Thus, we reused the data source, but the analytical datasets were different.

In conclusion, in this large population-based cohort study, fluoxetine use was not associated with an increased risk of acute pancreatitis compared with either citalopram or other SSRIs, and the absolute risk was low. Further research is needed to determine whether there is a class effect of SSRIs on the risk of acute pancreatitis.

## Ethics approval

The study was approved by the Danish Data Protection Agency through the University of Copenhagen (0421–0022/18–7000). Ethical approval is not required for register-based research in Denmark.

## Data availability

The data underlying this article cannot be shared publicly due to Danish data protection legislation of health care register data.

## Supplementary data

Supplementary data are available at *IJE* online.

## Author contributions

M.Aa. and M.An. obtained data for the study. All authors designed the study. M.Aa., S.K.B. and M.An. performed the analysis. M.Aa. wrote the first draft of the manuscript. All authors participated in the interpretation and discussion of the results, critically revised and approved the final version of the manuscript.

## Funding

The Pharmacovigilance Research Center is supported by a grant from the Novo Nordisk Foundation (NNF15SA0018404) to the University of Copenhagen.

## Conflict of interest

At the time of the study, M.L.D.B. was an employee of the Copenhagen Centre for Regulatory Science (CORS). CORS is a cross-faculty university-anchored institution involving various public (Danish Medicines Agency, Copenhagen University) and private (Novo Nordisk A/S, Lundbeck A/S, Ferring Pharmaceuticals A/S, LEO Pharma A/S) stakeholders as well as patient organiations (Rare Diseases Denmark). The centre is purely devoted to the scientific aspects of the regulatory field and has a patient-oriented focus, and the research is not a company-specific product or directly company related. Currently, M.L.D.B. is employed by Utrecht University to conduct research under the umbrella of the Utrecht Centre for Pharmaceutical Policy and Regulation. This centre receives no direct funding or donations from private parties, including the pharmaceutical industry. Research funding from public–private partnerships, e.g. IMI, the Escher Project [http://escher. lygat ure. org/], is accepted under the condition that no company-specific study is conducted. The centre has received unrestricted research funding from public sources, e.g. World Health Organization, Netherlands Organisation for Health Research and Development, the Dutch National Health Care Institute, EC Horizon 2020, the Dutch Medicines Evaluation Board and the Dutch Ministry of Health. M.An. has participated in research projects funded by AstraZeneca, H. Lundbeck & Mertz, Novartis and Pfizer, and has received fees for leading courses and teaching from Atrium, the Danish Association of the Pharmaceutical Industry. M.Aa., S.B.K., K.P., M.S. and K.P.D. have no conflicts of interest that are directly relevant to the content of this article.

## Supplementary Material

dyac071_Supplementary_Data
