# Supplementary material for: Investigation of the potential association between the use of fluoxetine and occurrence of acute pancreatitis: a Danish register-based cohort study
Source: Int J Epidemiol. 2022 Apr 26;51(5):1656–65. doi: 10.1093/ije/dyac071 (PMC9558065; doi:10.1093/ije/dyac071)
Supplement: dyac071_Supplementary_Data [file dyac071_supplementary_data.docx]

Supplementary material to: Investigation of the potential association between the use of fluoxetine and occurrence of acute pancreatitis: A Danish register-based cohort study

[Table S1 Definitions and time windows for exposures, outcomes, exclusion, and covariates 2](#_Toc90555211)

[Table S2 Primary and secondary analyses and their sensitivity analyses 3](#_Toc90555212)

[Figure S1 Hazard ratios and 95% confidence intervals (CIs) in the primary analysis comparing fluoxetine to citalopram and corresponding sensitivity analyses (S1-S8) 4](#_Toc90555213)

[Figure S2 Hazard ratios and 95% confidence intervals (CIs) in the secondary analysis comparing fluoxetine to other selective serotonin reuptake inhibitors and corresponding sensitivity analyses (S1-S8) 5](#_Toc90555214)

Corresponding author

Mia Aakjær, Pharmacovigilance Research Center, Department of Drug Design and Pharmacology, University of Copenhagen, Copenhagen, Denmark. E-mail: mia.aakjaer@sund.ku.dk.

| Table S1 Definitions and time windows for exposures, outcomes, exclusion, and covariates | | |
| --- | --- | --- |
| **Variable type** | **Code** | **Time window** |
| *Exposure* | | |
| Fluoxetine | ATC: N06AB03 | Index date |
| *Comparators* |  |  |
| Citalopram | ATC: N06AB04 | Index date |
| Other SSRIs | ATC: N06AB04, N06AB05, N06AB06, N06AB08, N06AB10 | Index date |
| *Outcomes* | |  |
| Acute pancreatitis | ICD-10: K85 | 6 and 3 months after the index date |
| Acute pancreatitis, sensitivity | ICD-10: K85.0, K85.3, K85.8, K85.9 | 6 and 3 months after the index date |
| *Exclusion* | |  |
| Acute pancreatitis | ICD-10: K85; ICD-8: 577.0 | 5 years before the index date and ever available in the data |
| Chronic pancreatitis | ICD-10: K86.0 K86.1; ICD-8: 577.1 | 5 years before the index date |
| Other pancreatic disorders | ICD-10: K86.2-K86.9; ICD-8: 577.9 | 5 years before the index date |
| Pancreatic cancer | ICD-10: C25; ICD-8: 157 | 5 years before the index date |
| SSRIs | ATC: N06AB | 1 year before the index date and 2 years before index date |
| *Covariates* | |  |
| Gallstones | ICD-10: K80; ICD-8: 574 | 5 years before the index date |
| Alcohol-related diseases and drug proxy | ICD-10: E24.4, F10, G31.2, G62.1, G72.1, I42.6, K29.2, K70, O35.4, T51; ICD-8: 291, 303, 571.09, 571.10, 456.0; ATC: N07BB | 5 years before the index date  6 months before the index date |
| Smoking-related lung diseases and drugs to treat COPD | ICD-10: J40-J44, J47, C34; ICD-8: 490-492, 518, 162  ATC: R03BA, R03AC, R03BB, R03CC | 5 years before the index date  6 months before the index date |
| Diabetes and antidiabetic medications | ICD-10: E10-E14; ICD-8: 249-250; ATC: A10 | 5 years before the index date  6 months before the index date |
| Obesity and anti-obesity medications | ICD-10: E66; ICD-8: 277  ATC: A08A | 5 years before the index date  6 months before the index date |
| Ischemic heart disease | ICD-10: I20-I25; ICD-8: 410-414 | 5 years before the index date |
| Inflammatory bowel disease | ICD-10: K50-K51; ICD-8: 563 | 5 years before the index date |
| Hyperlipidemia and statin use | ICD-10: E78; ICD-8: 272  ATC: C10AA | 5 years before the index date  6 months before the index date |
| HIV | ICD-10: B20-B24; ICD-8: 079.83 | 5 years before the index date |
| Endoscopic retrograde cholangiography (ERCP) | Procedure codes: 478.60, 478.70, 910.50, 911.25, KJKE02, KUJK02, KUJK05 | 5 years before the index date |
| Azathioprine and mercaptopurin (prodrug) | ATC: L04AX01, L01BB02 | 6 months before the index date |
| Calcium channel blockers | ATC: C08 | 6 months before the index date |
| NSAIDs excluding COX-2 | ATC: M01A excluding M01AX05 (glucosamine) and M01AH (coxibs) | 6 months before the index date |
| Valproic acid | ATC: N03AG01 | 6 months before the index date |
| ACE inhibitors | ATC: C09A, C09B | 6 months before the index date |
| Metronidazole | ATC: P01AB01 | 6 months before the index date |
| Oral glucocorticosteroids | ATC: H02AB | 6 months before the index date |

*ACE* angiotensin-converting enzyme*; ATC* Anatomical Therapeutic Chemical; *COPD* chronic obstructive pulmonary disease; *COX-2* cyclooxygenase-2; *HIV* human immunodeficiency virus; *ICD* International Statistical Classification of Diseases and Related Health Problems; *SSRIs* selective serotonin reuptake inhibitors

| Table S2 Primary and secondary analyses and their sensitivity analyses | | | | | |  |  |  |
| --- | --- | --- | --- | --- | --- | --- | --- | --- |
| Analysis | Outcome (ICD-10) | Follow-up (months) | Admission type | Diagnosis type | Recurrence | Washout of outcome (years) | Switching | Washout of exposure (years) |
| *Primary: Fluoxetine versus citalopram* | | | | | |  |  |  |
| Primary | K85 | 6 | Inpatients | A | No | 5 | No | 1 |
| S1, diagnosis type | K85 | 6 | Inpatients | A and B | No | 5 | No | 1 |
| S2, admission type | K85 | 6 | In- and outpatients | A | No | 5 | No | 1 |
| S3, outcome | K85.0, K85.3, K85.8, K85.9 | 6 | Inpatients | A | No | 5 | No | 1 |
| S4, follow-up | K85 | 3 | Inpatients | A | No | 5 | No | 1 |
| S5, recurrence | K85 | 6 | Inpatients | A | Yes | 5 | No | 1 |
| S6, washout of outcome | K85 | 6 | Inpatients | A | Yes | Ever | No | 1 |
| S7, switching | K85 | 6 | Inpatients | A | Yes | 5 | Yes | 1 |
| S8, washout of exposure | K85 | 6 | Inpatients | A | Yes | 5 | No | 2 |
| *Secondary: Fluoxetine versus SSRIs excl. fluoxetine* | | | | | |  |  |  |
| Secondary | K85 | 6 | Inpatients | A | No | 5 | No | 1 |
| S1, diagnosis type | K85 | 6 | Inpatients | A and B | No | 5 | No | 1 |
| S2, admission type | K85 | 6 | In- and outpatients | A | No | 5 | No | 1 |
| S3, outcome | K85.0, K85.3, K85.8, K85.9 | 6 | Inpatients | A | No | 5 | No | 1 |
| S4, follow-up | K85 | 3 | Inpatients | A | No | 5 | No | 1 |
| S5, recurrence | K85 | 6 | Inpatients | A | Yes | 5 | No | 1 |
| S6, washout of outcome | K85 | 6 | Inpatients | A | Yes | Ever | No | 1 |
| S7, switching | K85 | 6 | Inpatients | A | Yes | 5 | Yes | 1 |
| S8, washout of exposure | K85 | 6 | Inpatients | A | Yes | 5 | No | 2 |


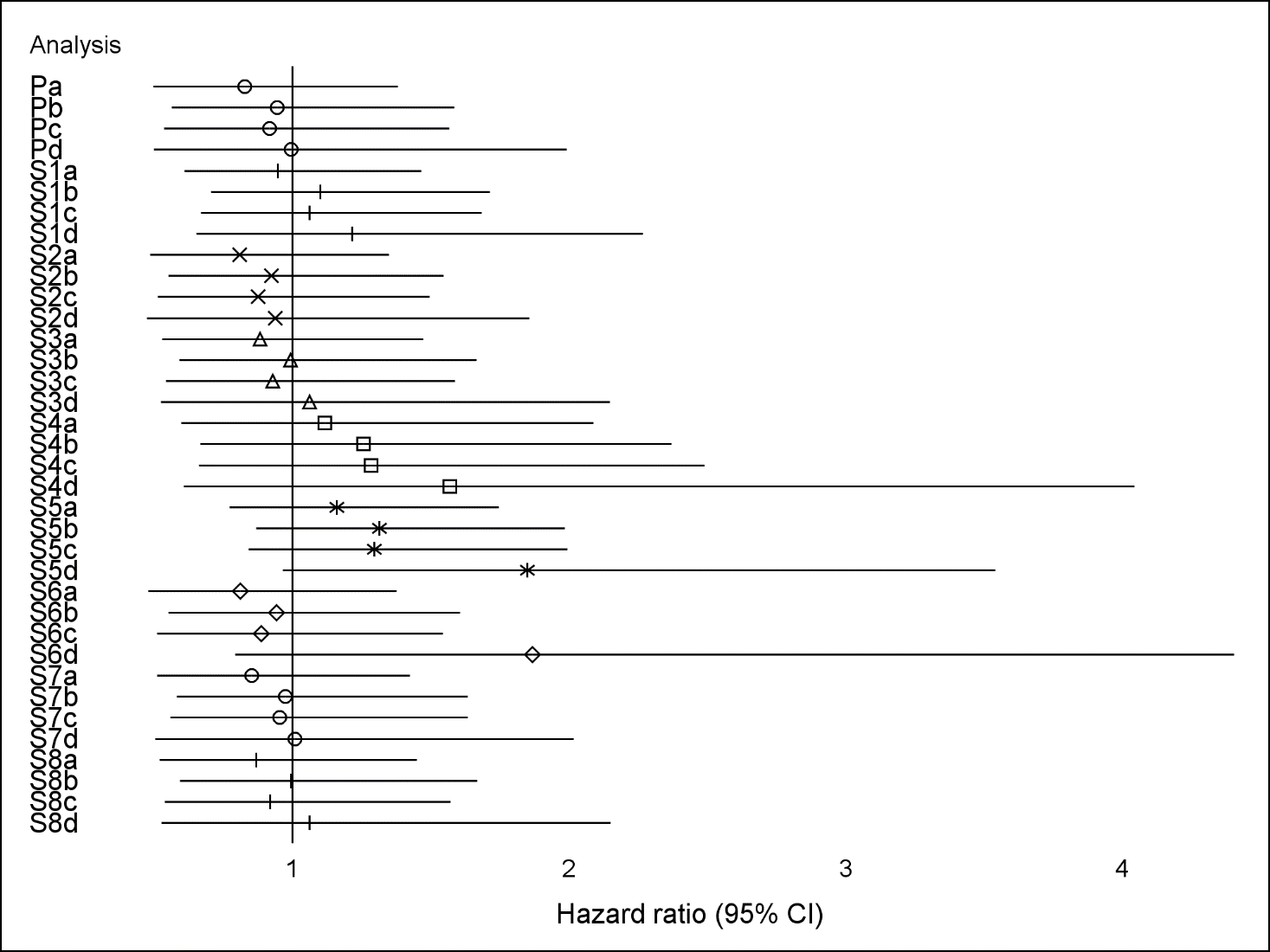


**Figure S1** Hazard ratios and 95% confidence intervals (CIs) in the primary analysis comparing fluoxetine to citalopram and corresponding sensitivity analyses (S1-S8); a: crude; b: age and sex; c: propensity score-adjusted; d: propensity score matched; P: primary; S1, diagnosis type: including secondary diagnoses in addition to the primary; S2, admission type: including outpatient visits in addition to inpatient hospitalization; S3, outcome: excluding ICD-10 codes representing gallstone and alcohol-induced acute pancreatitis; S4, follow-up: reduced from six to three months; S5, including recurrent acute pancreatitis events; S6, washout of outcome: increased from 5 years to ever available in the data; S7, switching: censoring at switching to another selective reuptake inhibitor; S8, washout of exposure, increased from 1 year to 2 years.


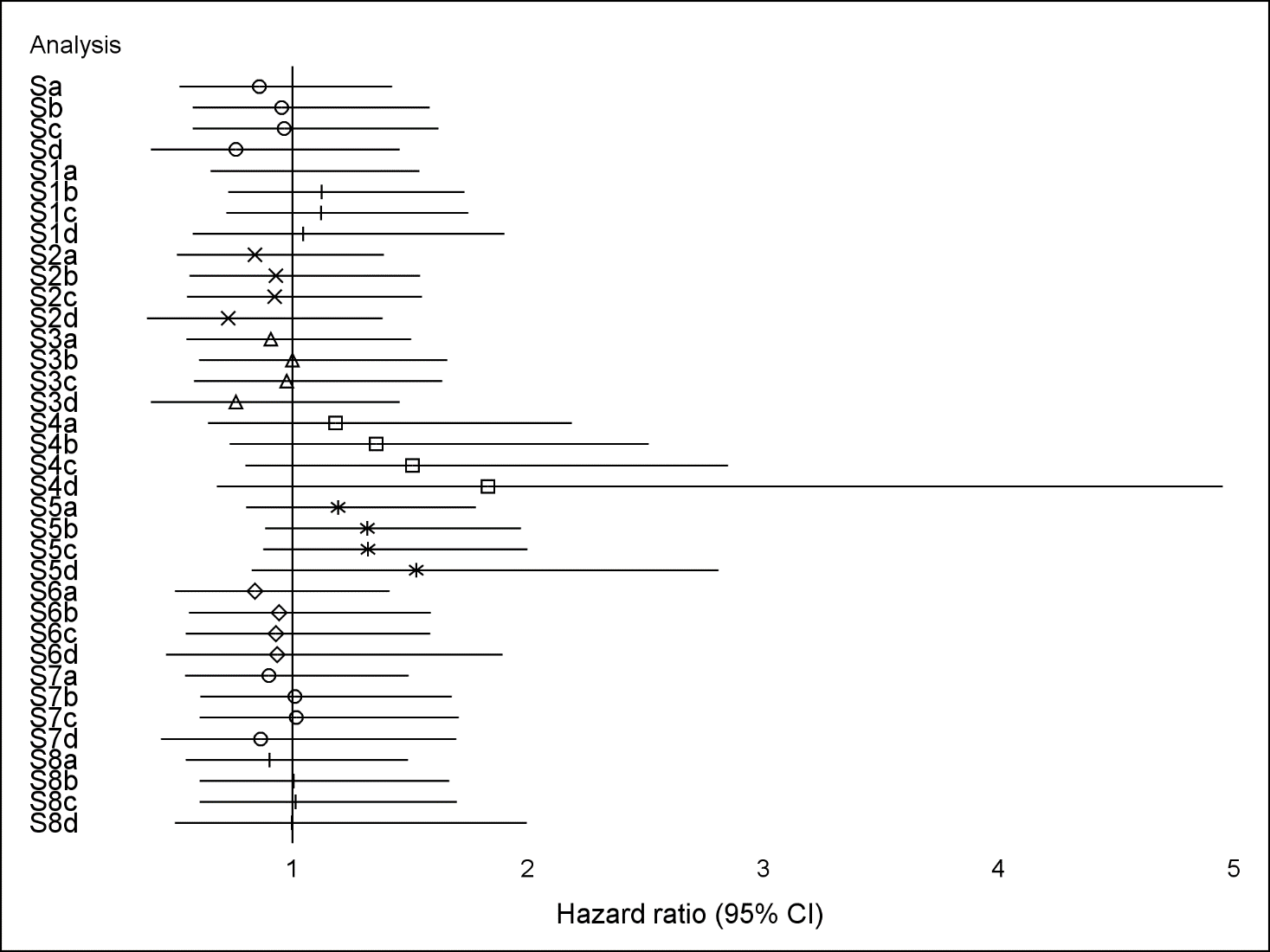


**Figure S2** Hazard ratios and 95% confidence intervals (CIs) in the secondary analysis comparing fluoxetine to other selective serotonin reuptake inhibitors and corresponding sensitivity analyses (S1-S8); a: crude; b: age and sex; c: propensity score-adjusted; d: propensity score matched; P: primary; S1, diagnosis type: including secondary diagnoses in addition to the primary; S2, admission type: including outpatient visits in addition to inpatient hospitalization; S3, outcome: excluding ICD-10 codes representing gallstone and alcohol-induced acute pancreatitis; S4, follow-up: reduced from six to three months; S5, including recurrent acute pancreatitis event; S6, washout of outcome: increased from 5 years to ever available in the data; S7, switching: censoring at switching to another selective reuptake inhibitor; S8, washout of exposure, increased from 1 year to 2 years.
